# Genomic characterization of *Lelliottia amnigena* PTJIIT1005, a nitrate tolerant strain isolated from water sample of Yamuna River, Delhi, India

**DOI:** 10.1128/mra.00229-22

**Published:** 2022-05-26

**Authors:** Preeti Thakur, Pammi Gauba

**Affiliations:** a Department of Biotechnology, Jaypee Institute of Information & Technology, Noida, Uttar Pradesh, India; Montana State University

## Abstract

Here, we report the genome sequence of PTJIIT1005, isolated from a polluted site on the Yamuna River, Delhi. The genome is complete and consists of ~4.5 Mbp with a GC content of 52.62%, 4,259 protein-coding genes, 76 tRNAs, and 4 rRNAs. Strain PTJIIT1005 shows 98.89% average nucleotide identity (ANI) with Lelliottia amnigena.

## ANNOUNCEMENT

The genus *Lelliottia* is composed of Gram-negative, rod-shaped gammaproteobacteria (1). Lelliottia amnigena (formerly referred to as Enterobacter amnigenus) members are facultatively anaerobic bacilli in nature that have been isolated from water and different food products and are known to cause bacterial infection in humans (2).

Lelliottia amnigena PTJIIT1005 was isolated from a water sample taken from the Yamuna River (Okhla Barrage, a polluted site), Delhi, India (28.565°N, 77.303°E), in May 2018. This strain was grown and maintained on nitrate agar medium at 30°C to 32°C under anaerobic conditions (3). *In vitro* analyses revealed that *L. amnigena* can grow at high nitrate concentrations.

Here, we report the complete genome sequence of *L. amnigena* to understand the molecular basis of nitrogen metabolism and the pathways involved in nitrate remediation of contaminated water.

Complete genome sequencing of the bacterium was performed at Redcliffe Laboratory (Noida, India). A single colony was inoculated into 5 mL nitrate broth and incubated at 32°C and 200 rpm in a shaker incubator. DNA was extracted using the NucleoSpin microbial DNA kit (Thermo Fisher Scientific) following the manufacturer’s instructions. Then, genomic libraries were prepared for sequencing using the QIAseq FX DNA library kit (Qiagen). Genome sequencing was performed using the Illumina NovaSeq 6000 platform. A paired-end (2 × 150-bp) sequencing library was prepared and then sequenced on the NovaSeq 6000 platform (Illumina). A total of 9,365,132 raw reads were obtained, providing 280× genome coverage. The fastp v0.20.1 tool was used to filter the reads. Of these, 8,596,940 Illumina reads were *de novo* assembled using Unicycler v0.4.4. The assembled genome sequence was annotated using the tool Prokka v1.12 (4). All tools were run with default parameters unless otherwise specified.

This genome sequence was assembled into 85 contigs comprising 4,552,797 bp, with a GC content of 52.62%. It contains 4,259 coding sequences, 76 tRNAs, 4 rRNAs, 1 transfer-messenger RNA (tmRNA), and 2,857 functional proteins. Details of the genome assembly can be found in Table 1.

**TABLE 1:** Assembly details

Characteristic	Value
BioProject accession no. (raw reads)	PRJNA830830
BioSample accession no. (raw reads)	SAMN27738499
GenBank assembly accession no.	JAKRZN010000000
Total no. of Illumina raw reads	8,596,940
Estimated genome coverage (×)	280
Assembler used	Unicycler v0.4.4
Total no. of scaffolds obtained	85
Total size of the scaffolds (bp [Mb])	4,552,797 (~4.5)
Minimum scaffold length (bp)	100
Maximum scaffold length (bp)	1,125,792
Avg scaffold length (bp)	53,562.3
No. of protein coding genes	4,259
GC content (%)	52.92
No. of tRNAs	76

The RAST tool (5) was used for functional analysis, which revealed 104 metabolism subsystems, in which 34 genes accounted for nitrogen metabolism (including nitrate and nitrite utilization).

This strain has nitrate remediation traits that can contribute to the removal of nitrate from water.

The average nucleotide identity (ANI) (6) analysis of PTJIIT1005 with closely related species revealed a similarity index of 98.89% with Lelliottia amnigena (GenBank accession number NZ_CP077236.1).

### Data availability.

This whole-genome shotgun project has been deposited at DDBJ/ENA/GenBank under accession number JAKRZN000000000 (BioProject accession number PRJNA806758, BioSample accession number SAMN25898493). The Illumina raw reads have also been deposited at the Sequence Read Archive (SRA) under BioProject accession number PRJNA830830 and BioSample accession number SAMN27738499.
